# Supplementary material for: Rapid Detection of Antimicrobial Resistance Genes in Critically Ill Children Using a Custom TaqMan Array Card
Source: Antibiotics (Basel). 2023 Dec 5;12(12):1701. doi: 10.3390/antibiotics12121701 (PMC10740637; doi:10.3390/antibiotics12121701)
Supplement: Supplementary file 1 [file antibiotics-12-01701-s001.zip › antibiotics-2722867-supplementary.pdf]

Supplementary Tables and Figures - Rapid Detection of Antimicrobial Resistance Genes in Critically Ill Children Using a Custom TaqMan Array Card

John A. Clark [1,2,*], Martin D. Curran [3], Theodore Gouliouris [2,3], Andrew Conway Morris [2,4,5], Rachel Bousfield [2,3], Vilas Navapurkar [2], Iain R. L. Kean [1], Esther Daubney [1], Deborah White [1], Stephen Baker [6] and Nazima Pathan [1,2]

1. Department of Paediatrics, University of Cambridge, Cambridge CB2 1TN, UK; irlk2@cam.ac.uk (I.R.L.K.); esther.daubney@nhs.net (E.D.); deborah.white31@nhs.net (D.W.); np409@cam.ac.uk (N.P.)
2. Cambridge University Hospitals NHS Foundation Trust, UK; theodore.gouliouris@nhs.net (T.G.); ac926@cam.ac.uk (A.C.M.); rachel.bousfield@nhs.net (R.B.); vilas.navapurkar@nhs.net (V.N.)
3. United Kingdom Health Security Agency, Clinical Microbiology and Public Health Laboratory,
Cambridge, UK; martin.curran@nhs.net
4. Division of Anaesthesia, Department of Medicine, University of Cambridge, Cambridge CB2 1TN, UK
5. Division of Immunology, Department of Pathology, University of Cambridge, Cambridge CB2 1TN, UK
6. Cambridge Institute of Therapeutic Immunology and Infectious Disease, University of Cambridge,
Cambridge CB2 1TN, UK; sgb47@cam.ac.uk

* Correspondence: jacla7@gmail.com; Tel.: +61-(0)-43725-6450

Table S1: Targets on the antimicrobial resistance gene TaqMan array card

AMR gene target	Assay name	Assay number	Forward primer 1000nM	Reverse primer 1000nM	6FAM MGB probe 250nM
bla$_{\text{CTX-M-1}}$	Ba04646149_s1	10, 34			
	N/A	22	ATGTGCAGYACCAGTAARGTKATGGC	ATCACKCGGRTCGCCIGGRAT	CCCGACAGCTGGGAGACGAAACGT
bla$_{\text{CTX-M-2}}$	Ba04646142_s1	12, 35			
bla$_{\text{CTX-M-8/25}}$	Ba04646154_s1	13, 36			
bla$_{\text{CTX-M-9}}$	Ba04646127_s1	14, 37			
bla$_{\text{CTX-M}}$	N/A	23	ATGTGCAGYACCAGTAARGTKATGGC	ATCACKCGGRTCGCCIGGRAT	CGAYAATACNGCCATGAA
ermB	Pa04230913_s1	15, 38			
bla$_{\text{FOX}}$ *(ampC)*	Ba04646126_s1	2, 26			
bla$_{\text{GES}}$	Ba04646151_s1	3, 27			
bla$_{\text{IMP}}$	N/A	47	CCCACGTATGCATCTGWATTAACAAA	CCAAACCACTACGTTATCTKGAGTG	AAGACGGTAAGGTNCAAGCYA
	N/A	48	CCCACGTATGCATCTGWATTAACAAA	CCAAACCACTACGTTATCTKGAGTG	AATCCGGTAAGGTNCAAGCYA
bla$_{\text{IMP-1}}$	Ba04646131_s1	5, 29			
bla$_{\text{KPC}}$	Ba04646152_s1	4, 28			
	N/A	44	GCAGCGGCAGCAGTTTGTTGATT	GTAGACGGCCAACACAATAGGTGC	CAGTCGGAGACAAAACCGGA
mecA	N/A	20	TGGTATGTGGAAGTTAGATTGGGAT	AGATACATTCTTTGGAACGATGCCTA	CCAGGAATGCAGAAAGACCA
mecA/C	N/A	21	CAAGCAATAGAATCATCAGAYAACAT	CCRTAACCTGAATCWGCTAATAATATTTC	CCRAGTGATTATCCMTT
bla$_{\text{NDM}}$	N/A	46	CAGCAAATGGAAACTGGCGAC	ATCCAGTTGAGGATCTGGGC	CCGAATGTCTGGCAGCACACT
bla$_{\text{NDM-1}}$	Ba04931076_s1	6, 30			
bla$_{\text{OXA-48}}$	N/A	43	ATTCGGGCTAAAACKGGATACTCGRCT	GATGTGGGCATATCCATATTCATCGCA	ACCAGCCAATCTTAGGTTCGAT
	Ba04930816_s1	7, 31			
bla$_{\text{OXA-1}}$	Ba04646133_s1	1, 25			
bla$_{\text{PER-1}}$	Ba04646140_s1	8, 32			
qnrA	Ba04646160_s1	16, 39			
qnrS	Ba04646145_s1	17, 40			
vanA	N/A	24	GGGCAGAGTATTGACTTCGT	CCCGTGTGGATATGTTTTTACAAG	CCGCATTGTACTGAACG
vanB	N/A	42	TCCGGTCGAGGAACGAAA	GCCCTCTGCATCCAAGCA	ACGGCAAAGAAAGTATATCGG
bla$_{\text{VEB}}$	Ba04646153_s1	9, 33			
bla$_{\text{VIM}}$	Ba04646155_s1	18, 41			
	N/A	45	GCAAATTGGACTTCCYGTAACGC	CCCGAAGGACATCAACGCC	ACGCACTTTCATGACGACCG
18S rRNA gene	N/A	11			
MS2	N/A	19	TGGCACTACCCCTCTCCGTATTCACG	GTACGGGCGACCCCACGATGAC	TCGATAGATCAAGGTGCCT

Table S2: Validation of antimicrobial resistance gene TaqMan array card with plasmids and ZeptoMetrix NATtrol panel

Validation	AMR genes present in validation sample	AMR-TAC Ct value(s)
VAP plasmid 1	$bla_{CTX\text{-}M\text{-}1}$	$bla_{CTX\text{-}M\text{-}1}$: 24/24
	$bla_{OXA\text{-}48}$	$bla_{CTX\text{-}M}$: 27
	bla_{IMP}	$bla_{OXA\text{-}48}$: 26/27/26
		bla_{IMP}: 26/25
VAP plasmid 2	$bla_{CTX\text{-}M}$	$bla_{CTX\text{-}M\text{-}9}$: 27/28
	bla_{NDM}	bla_{NDM}: 27
	vanA	*vanA:* 26
	bla_{VIM}	bla_{VIM}: 32/28/21
VAP plasmid 3	bla_{KPC}	bla_{KPC}: 19
	mecC	*mecA*/*C*: 25
	vanB	*vanB*: 18
ZeptoMetrix pool 1	bla_{IMP}	$bla_{IMP\text{-}1}$: 30/31
	$bla_{OXA\text{-}48\text{-}like}$	bla_{IMP}: 33
	$bla_{CTX\text{-}M}$	$bla_{OXA\text{-}1}$: 22/22
		$bla_{OXA\text{-}48}$: 22/23/23
		$bla_{CTX\text{-}M\text{-}1}$: 22.5/22
		$bla_{CTX\text{-}M}$: 25
ZeptoMetrix pool 2	bla_{KPC}	bla_{KPC}: 24/25/21
	bla_{VIM}	$bla_{OXA\text{-}1}$: 27/27
		bla_{VIM}: 28/30/28
ZeptoMetrix pool 3	bla_{NDM}	$bla_{NDM\text{-}1}$: 22/22
	$bla_{CTX\text{-}M}$	bla_{NDM}: 21
	mecA/*C*, MREJ	$bla_{OXA\text{-}1}$: 24/23
		$bla_{CTX\text{-}M\text{-}1}$: 20/19
		$bla_{CTX\text{-}M}$: 23
		mecA: 27
		mecA/*C*: 28
ZeptoMetrix pool 4	N/A	Nil
ZeptoMetrix pool 5	N/A	Nil

Table S3: Inter-assay reproducibility of an antimicrobial resistance gene TaqMan array card using faecal sewage

**Assay**		**Cycle threshold value**								
		**Card 1**	**Card 2**	**Card 3**	**Card 4**	**Card 5**	**Card 6**	**Card 7**	**Mean**	**(SD)**
1	$bla_{\text{OXA-1}}$	21.4	21.0	22.1	23.8	23.2	24.6	21.7	22.6	(1.3)
2	*ampC*	24.5	24.2	24.7	27.2	26.4	28.3	24.7	25.7	(1.6)
3	bla_{GES}	19.1	19.0	19.7	21.1	20.3	21.8	19.2	20.0	(1.1)
4	bla_{KPC}	26.0	25.9	26.4	26.0	26.3	27.4	25.5	26.2	(0.6)
5	$bla_{\text{IMP-1}}$	22.3	22.3	23.4	24.3	24.9	24.8	22.5	23.5	(1.2)
6	$bla_{\text{NDM-1}}$	22.2	21.5	22.3	24.3	23.9	24.7	21.5	22.9	(1.3)
7	$bla_{\text{OXA-48}}$	26.2	25.5	27.4	27.6	27.0	27.8	26.1	26.8	(0.9)
8	$bla_{\text{PER-1}}$	24.6	24.6	25.1	25.9	24.9	25.6	25.2	25.1	(0.5)
9	bla_{VEB}	25.1	25.5	26.4	27.1	26.9	28.1	25.5	26.4	(1.1)
10	$bla_{\text{CTX-M-1}}$	28.3	28.0	28.4	26.5	26.5	26.6	27.7	27.4	(0.9)
11	18S rRNA gene	16.6	16.5	17.0	16.7	15.6	16.3	15.6	16.3	(0.5)
12	$bla_{\text{CTX-M-2}}$	-	-	-	-	-	-	-		
13	$bla_{\text{CTX-M-8/25}}$	-	-	-	-	-	-	-		
14	$bla_{\text{CTX-M-9}}$	33.3	32.4	34.9	33.4	33.1	34.5	31.6	33.3	(1.1)
15	*ermB*	20.9	21.5	22.8	21.7	21.1	21.6	21.1	21.5	(0.6)
16	*qnrA*	23.9	23.3	24.2	26.7	26.3	27.1	23.2	25.0	(1.7)
17	*qnrS*	35.3	33.5	33.6		34.0	34.5	32.5	33.9	(1.0)
18	bla_{VIM}	26.5	27.0	30.4	27.3	25.2	27.5	26.4	27.2	(1.6)
19	MS2	29.7	29.4	31.0	30.0	27.2	30.5	27.6	29.3	(1.4)
20	*mecA*	30.9	-	36.2	32.7	-	32.2	-	33.0	(2.3)
21	*mecA/C*	34.8	35.0	36.7	33.3	35.9	33.8	33.9	34.8	(1.2)
22	$bla_{\text{CTX-M}}$	31.4	32.1	33.6	27.9	29.5	29.6	30.5	30.7	(1.9)
23	$bla_{\text{CTX-M}}$	!	!	!	!	!	!	!		
24	*vanA*	25.3	26.4	27.0	26.3	25.8	26.1	25.8	26.1	(0.5)
25	$bla_{\text{OXA-1}}$	21.2	21.4	22.2	23.7	23.2	24.4	22.0	22.6	(1.2)
26	*ampC*	25.1	24.8	25.5	27.9	26.5	28.4	24.6	26.1	(1.5)
27	bla_{GES}	18.3	18.5	19.6	20.7	20.5	21.6	19.3	19.8	(1.2)
28	bla_{KPC}	26.1	26.1	23.4	26.2	26.3	27.5	25.3	25.9	(1.3)
29	$bla_{\text{IMP-1}}$	23.2	22.7	23.6	25.4	24.9	25.0	22.6	23.9	(1.2)
30	$bla_{\text{NDM-1}}$	22.3	22.2	22.1	24.7	24.1	24.4	21.6	23.1	(1.3)
31	$bla_{\text{OXA-48}}$	25.6	26.9	28.6	27.8	26.9	28.1	26.7	27.2	(1.0)
32	$bla_{\text{PER-1}}$	25.0	24.9	25.3	25.6	25.2	25.8	25.2	25.3	(0.3)
33	bla_{VEB}	25.7	25.6	26.1	27.5	26.8	27.4	25.2	26.3	(0.9)
34	$bla_{\text{CTX-M-1}}$	28.4	28.1	28.3	26.4	26.4	26.2	27.5	27.3	(1.0)
35	$bla_{\text{CTX-M-2}}$	-	-	-	-	-	-	-		
36	$bla_{\text{CTX-M-8/25}}$	-	-	-	-	-	-	-		
37	$bla_{\text{CTX-M-9}}$	34.4	33.5	33.7	35.4	35.6	33.0	32.7	34.0	(1.2)
38	*ermB*	21.6	21.4	21.7	21.6	21.3	22.3	21.0	21.6	(0.4)
39	*qnrA*	23.8	23.3	23.5	26.4	26.5	26.4	23.1	24.7	(1.6)
40	*qnrS*	36.6	35.0	36.1		37.7	34.9	33.1	35.6	(1.6)
41	bla_{VIM}	25.7	27.2	29.4	28.6	25.1	27.1	26.1	27.0	(1.5)
42	*vanB*	25.7	27.7	29.0	24.9	24.8	25.9	27.4	26.5	(1.6)

43	*bla*OXA	22.4	21.1	22.5	23.6	23.1	24.3	21.8	22.7	(1.1)
44	*bla*KPC	27.1	26.6	26.7	27.4	26.2	26.0	25.6	26.5	(0.6)
45	*bla*VIM	23.6	22.8	23.1	23.5	22.1	23.0	22.2	22.9	(0.6)
46	*bla*NDM	24.7	21.4	24.4	27.7	26.1	27.6	23.9	25.1	(2.2)
47	*bla*IMP	24.9	23.9	26.7	26.5	27.5	28.3	26.0	26.3	(1.5)
48	*bla*IMP	26.3	31.5	33.2	30.0	31.9	30.8	29.9	30.5	(2.2)

! :failed assay; - :negative

Table S4: Antimicrobial resistance genes identified on samples with phenotypic resistance

Validation sample	Sample ID	Bacteria	Known phenotypic/ genotypic resistance	AMR gene card result	Concordant with AMR-TAC
1	102074	*S. aureus*	MRSA	*mecA/C*: 16	◎
2	102073	*S. aureus*	MRSA	*mecA/C*: 16	◎
3	102072	*S. aureus*	MRSA	*mecA*: 31<br>*mecA/C*: 32	◎
4	SA227	*S. aureus*	*MecC*	*mecA/C*: 16	◎
5	LGA 706(2)	*S. aureus*	*MecC*	*mecA/C*: 21	◎
6	160002 MU50	*S. aureus*	MSSA	-	◎
7	73048 eMRSA 3	*S. aureus*	MRSA	*mecA*: 15<br>*mecA/C*: 15	◎
8	17077 France 5	*S. aureus*	MRSA	*mecA*:13<br>*mecA/C*: 15	◎
9	17098 CA - MRSA MLST ST80	*S. aureus*	MRSA	*mecA*: 15<br>*mecA/C*: 16	◎
10	07045 Australia Sydney	*S. aureus*	MRSA	*mecA*: 13<br>*mecA/C*: 15	◎
11	WIS21093 19	*S. aureus*	MRSA	*mecA*: 14<br>*mecA/C*: 15	◎
12	17061 Japan 8	*S. aureus*	MRSA	*mecA*: 15<br>*mecA/C*: 16<br>*ermB*: 15/14	◎/?
13	NCTC 13379	*E. faecalis*	*vanHBX*<br>*ermB*<br>*aac(6′)-aph(2″)*[$]<br>*ant(6)-la*[$]<br>*aph(3′)-III*[$]<br>*Isa(A)*[$]<br>*qacZ*[$]<br>*cat(pC233)*[$]	*vanB*: 16<br>*ermB*: 17/16	◎
14	NCTC 12952	*E. faecium*	*vanHBX*<br>*msr(C)*[$]<br>*tet(M)*[$]<br>*aac(6′)-li*[$]	*vanB*: 17	◎
15	73082 France 2	*S. aureus*	MRSA	*mecA*:16<br>*mecA/C*: 17	◎
16	73047 - 4	*S. aureus*	MRSA	*mecA*: 13<br>*mecA/C*: 15	◎
17	17069 Denmark DLL4916 - 7	*S. aureus*	MRSA	*mecA*: 16<br>*mecA/C*: 18	◎
18	17048 UK early strain RL13136	*S. aureus*	MRSA	*mecA*: 15<br>*mecA/C*: 18	◎
19	07060 Atlanta USA 80760R7	*S. aureus*	MRSA	*mecA*: 18<br>*mecA/C*: 18	◎
20	07059 - SF40R6LA	*S. aureus*	MRSA	*mecA*: 15<br>*mecA/C*: 16	◎
21	* Anonymised *VRE	*E. faecium*	*vanA*	*vanA*: 20<br>*ermB*: 18/18	◎/?
22	* Anonymised * VRE	*E. faecium*	*vanA*	*vanA*: 22<br>*ermB*: 20/20	◎/?
23	* Anonymised * VRE	*E. faecium*	*vanA*	*vanA*: 21	◎

24	* Anonymised *	*E. faecium*	*vanA*	*vanA*: 22<br>*ermB*: 19/19	🌀/?
25	* Anonymised *	*E. faecalis*	*vanA*	*vanA*: 19<br>*ermB*: 18/18	🌀/?
26	* Anonymised *	*E. faecalis*	*vanA*	*vanA*: 20<br>*ermB*: 19/19	🌀/?
27	* Anonymised *	*E faecium*	Fully susceptible	-	🌀
28	NCTC 13439	*K. pneumoniae*	*qnrS1*<br>*bla*$_{VIM-1}$<br>*fosA*$^{\$}$<br>*aac(6')-Ib3*$^{\$}$<br>*aac(6')-Ib-cr*$^{\$}$<br>*OqxB*$^{\$}$<br>*OqxA*$^{\$}$<br>*dfrA14*$^{\$}$<br>*blaSHV-178*$^{\$}$<br>*bla*$_{SHV-36}$$^{\$}$<br>*bla*$_{SHV-80}$$^{\$}$<br>*bla*$_{SHV-12}$$^{\$}$	*qnrS*: 21/22<br>*bla*$_{VIM}$: 28/28/23	🌀

29	NCTC 13443	*K. pneumoniae*	*bla*$_{NDM-1}$<br>*bla*$_{CTX-M-15}$<br>*bla*$_{OXA-1}$<br>*msr(E)*$^{\$}$<br>*fosA*$^{\$}$<br>*aac(3)-Iid*$^{\$}$<br>*aph(6)-Id*$^{\$}$<br>*aac(6')-Ib*$^{\$}$<br>*aadA1*$^{\$}$<br>*aac(6')-Ib-cr*$^{\$}$<br>*aac(3)-IIa*$^{\$}$<br>*armA, aph(3")-Ib*$^{\$}$<br>*ere(A)*$^{\$}$<br>*msr(E)*$^{\$}$<br>*mph(E)*$^{\$}$<br>*OqxA*$^{\$}$<br>*OqxB*$^{\$}$<br>*dfrA1*$^{\$}$<br>*sul2*$^{\$}$<br>*sul1*$^{\$}$<br>*ARR-2*$^{\$}$<br>*aadA1*$^{\$}$<br>*bla*$_{SHV-70}$$^{\$}$<br>*bla*$_{SHV-13}$$^{\$}$<br>*bla*$_{SHV-11}$$^{\$}$<br>*bla*$_{SHV-28}$$^{\$}$<br>*bla*$_{SHV-25}$$^{\$}$ *bla*$_{TEM-1A}$$^{\$}$ *bla*$_{DHA-7}$$^{\$}$<br>*bla*$_{SHV-31}$$^{\$}$<br>*bla*$_{DHA-24}$$^{\$}$<br>*bla*$_{SHV-69}$$^{\$}$<br>*bla*$_{SHV-185}$$^{\$}$<br>*bla*$_{CMY-4}$ $^{\$}$<br>*bla*$_{DHA-1}$$^{\$}$<br>*bla*$_{SHV-106}$$^{\$}$ *bla*$_{TEM-1B}$$^{\$}$<br>*qacE*$^{\$}$<br>*catB3*$^{\$}$<br>*catA1*$^{\$}$<br>*cmlA1*$^{\$}$	*bla*$_{NDM-1}$: 17/17<br>*bla*$_{NDM}$: 19<br>*bla*$_{CTX-M}$: 18<br>*bla*$_{CTX-M-1}$: *17/18*<br>*qnrS*: 18/18	◎/*x*

30	NCTC 13442	*K. pneumoniae*	*bla*$_{OXA\text{-}48}$<br>*aph(3′)-Ia*$^{\$}$<br>*fosA*$^{\$}$<br>*ere(A)*$^{\$}$<br>*OqxA*$^{\$}$<br>*OqxB*$^{\$}$<br>*dfrA5*$^{\$}$<br>*sul1*$^{\$}$<br>*dfra14*$^{\$}$<br>*tet(C)*$^{\$}$<br>*bla*$_{SHV\text{-}79}$$^{\$}$<br>*bla*$_{SHV\text{-}89}$$^{\$}$<br>*bla*$_{SHV\text{-}56}$$^{\$}$<br>*bla*$_{SHV\text{-}85}$$^{\$}$<br>*bla*$_{TEM\text{-}1B}$$^{\$}$<br>*bla*$_{SHV\text{-}4}$$^{\$}$<br>*qacE*$^{\$}$<br>*catA1*$^{\$}$	*bla*$_{OXA\text{-}48}$: 16/18/17	◎
31	NCTC 13438	*K. pneumoniae*	*bla*$_{KPC\text{-}3}$<br>*bla*$_{OXA\text{-}9}$$^{\$}$<br>*aph(3′)-Ia*$^{\$}$<br>*aac(6′)-Ib*$^{\$}$<br>*aadA2b*$^{\$}$<br>*aadA2*$^{\$}$<br>*fosA*$^{\$}$<br>*mph(A)*$^{\$}$<br>*OqxA*$^{\$}$<br>*OqxB*$^{\$}$<br>*aac(6′)-Ib-cr*$^{\$}$<br>*sul1*$^{\$}$<br>*dfrA12*$^{\$}$<br>*bla*$_{SHV\text{-}182}$$^{\$}$<br>*bla*$_{TEM\text{-}1A}$$^{\$}$<br>*qacE*$^{\$}$<br>*catA1*$^{\$}$	*bla*$_{KPC}$: 18/17/19	◎

32	* Anonymised *	*E. cloacae*	*bla*$_{IMP}$	*bla*$_{IMP}$: 20<br>*bla*$_{IMP-1}$: 16/16<br>*qnrA*: 16/16	◎/?
33	* Anonymised *	*K. pneumoniae*	*bla*$_{NDM}$	*bla*$_{NDM}$: 17<br>*bla*$_{NDM-1}$: 15/16<br>*bla*$_{CTX-M}$: 18<br>*bla*$_{CTX-M-1}$: 17/17	◎/?
34	* Anonymised *	*E. cloacae*	*bla*$_{IMP-1}$	*bla*$_{IMP}$: 18<br>*bla*$_{IMP-1}$: 15/15<br>*qnrA*: 15/15	◎/?
35	ME21	Unknown	*bla*$_{KPC}$<br>*bla*$_{OXA-48}$	*bla*$_{KPC}$: 18/19/18<br>*bla*$_{OXA-48}$: 35/34/18<br>*bla*$_{CTX-M-9}$: 20/20<br>*qnrA*: 18/18<br>*qnrS*: 18/17	◎/?
36	* Anonymised *	*P. aeruginosa*	Unknown	-	?
37	* Anonymised *	*P. aeruginosa*	Fully susceptible	-	◎
38	* Anonymised *	*P. aeruginosa*	Fully susceptible	-	◎
39	ESBL15-01	*S. marcescens*	*bla*$_{IMP-1}$	*bla*$_{IMP}$: 16/18<br>*bla*$_{IMP-1}$: 19/18	◎
40	ESBL15-02	*E. coli*	*bla*$_{OXA-48}$<br>*bla*$_{CTX-M-27 \text{ (group 9)}}$<br>*bla*$_{TEM-1}$ $	*bla*$_{OXA-48}$: 18/21/20<br>*bla*$_{CTX-M-9}$: 20/22<br>*ermB*: 20/20	◎/?
41	ESBL15-04	*K. pneumoniae*	*bla*$_{OXA-232}$ (*bla*$_{OXA-48-like}$)<br>*bla*$_{NDM-1}$<br>*bla*$_{SHV-28}$ $	*bla*$_{NDM-1}$: 22/22<br>*bla*$_{NDM}$: 25<br>*bla*$_{OXA-1}$: 22/22<br>*bla*$_{OXA-48}$: 20	◎
42	ESBL15-05	*E. cloacae*	*bla*$_{CTX-M-15 \text{(group 1)}}$<br>*bla*$_{NDM-1}$<br>*bla*$_{TEM-1}$ $<br>*bla*$_{SHV-12}$ $	*bla*$_{OXA-1}$: 19/18<br>*bla*$_{CTX-M}$: 18<br>*bla*$_{CTX-M-1}$: *16/17*<br>*bla*$_{NDM}$: 20<br>*bla*$_{NDM-1}$: 18/17	◎/?
43	ESBL15-07	*E. coli*	*bla*$_{CTX-M-55 \text{(group 1)}}$<br>*bla*$_{TEM-1}$ $	*bla*$_{CTX-M}$: 21<br>*bla*$_{CTX-M-1}$: 20/20<br>*bla*$_{NDM}$: 20<br>*bla*$_{NDM-1}$: 18/17	◎/?
44	ESBL15-09	*E. coli* / *K. pneumoniae*	*bla*$_{KPC-3}$<br>*bla*$_{OXA-48}$<br>*bla*$_{CTX-M-27 \text{(group 9)}}$<br>*bla*$_{TEM-1}$ $<br>*bla*$_{SHV-11}$ $	*bla*$_{KPC}$: 23/23/24<br>*bla*$_{OXA-48}$: 21/19/21<br>*bla*$_{CTX-M-9}$: 22/22<br>*ermB*: 21/21	◎/?
45	ESBL15-10	*Enterobacter aerogenes*	*bla*$_{TEM-24}$ $<br>*bla*$_{SHV-2a}$ $		◎
46	ESBL15-11	*K. pneumoniae*	*bla*$_{CTX-M-14 \text{(group 9)}}$<br>*bla*$_{SHU-76-like}$ $	*bla*$_{CTX-M-9}$: 22/23<br>*qnrS*: 21/22	◎/?
47	GES control (neat)	Unknown	*bla*$_{GES}$	*bla*$_{GES}$: 15/14	◎

48	GES control (1:20)	Unknown	bla_{GES}	bla_{GES}: 13/11	◎
49	FOX control (neat)	Unknown	*AmpC*	*AmpC*: 20/10	◎
50	FOX control (1:20)	Unknown	*AmpC*	*AmpC*: 23/23	◎
51	$bla_{\text{CTX-M-15}}$ control (neat)	Unknown	$bla_{\text{CTX-M-15}}$	$bla_{\text{OXA-1}}$: 20/21<br>$bla_{\text{CTX-M}}$: 21<br>$bla_{\text{CTX-M-1}}$: 18/19	◎/?

52	*bla*CTX-M-2 control (neat)	Unknown	*bla*CTX-M-2	*bla*CTX-M-2: 22/22	◎
53	*bla*CTX-M-9 control (neat)	Unknown	*bla*CTX-M-9	*bla*CTX-M-9: 21/22<br>*qnrA*: 22/21	◎/?
54	*bla*CTX-M-14 control (neat)	Unknown	*bla*CTX-M-14	*bla*CTX-M-9: 20/22	◎
55	3* Anonymised *	*E. faecium*	VRE: ampicillin and vancomycin resistant	*vanA*: 21<br>*ermB*: 21/21	◎/?
56	* Anonymised *	*E. faecium*	VRE: ampicillin and vancomycin resistant	*vanA*: 22<br>*ermB*: 20/22	◎/?
57	NCTC 13437	*P. aeruginosa*	*bla*VIM-2<br>*bla*VEB-1<br>*fosA*[$]<br>*aadA1*[$]<br>*aph(3')-Iib*[$]<br>*aac(6')-II*[$]<br>*ant(2")-Ia*[$]<br>*aac(6')-II*[$]<br>*crpP, sul1*[$]<br>*dfrB2*[$]<br>*tet(A)*[$]<br>*bla*PAO[$]<br>*bla*OXA-50[$]<br>*bla*OXA-10[$]<br>*qacE*[$]<br>*catB7*[$]	*bla*VIM: 15/19/25<br>*bla*VEB: 17/16	◎
58	NCTC 13443	*K. pneumoniae*	*bla*NDM-1<br>*bla*OXA-1<br>*bla*CTX-M-15<br>*msr(E)*[$]<br>*fosA*[$]<br>*aac(3)-Iid*[$]<br>*aph(6)-Id*[$]<br>*aac(6')-Ib*[$]<br>*aadA1*[$]<br>*aac(6')-Ib-cr*[$]<br>*aac(3)-IIa*[$]<br>*armA*[$]<br>*aph(3")-Ib*[$]<br>*ere(A)*[$]<br>*msr(E)*[$]<br>*mph(E*[$]<br>*OqxA*[$]<br>*OqxB*[$]<br>*dfrA1*[$]<br>*sul2*[$]<br>*sul1*[$]<br>*ARR-2*[$]<br>*aadA1*[$]<br>*bla*SHV-70[$]<br>*bla*SHV-13[$]<br>*bla*SHV-11[$]<br>*bla*SHV-28[$]<br>*bla*SHV-25[$]<br>*bla*TEM-1A[$]	*bla*NDM: 23/22/25<br>*bla*OXA-1: 20/20<br>*bla*CTX-M-1: 17/17/16	◎

			$bla_{\text{DHA-7}}^{\$}$<br>$bla_{\text{SHV-31}}^{\$}$<br>$bla_{\text{DHA-24}}^{\$}$<br>$bla_{\text{SHV-69}}^{\$}$<br>$bla_{SHV\text{-}185}^{\$}$<br>$bla_{\text{CMY-4}}^{\$\$}$<br>$bla_{\text{DHA-1}}^{\$}$<br>$bla_{\text{SHV-106}}^{\$}$<br>$bla_{\text{TEM-1}}^{\$}$<br>*qacE*$^{\$}$<br>*qacE*$^{\$}$<br>*catB3*$^{\$}$<br>*catA1*$^{\$}$<br>*cmlA1*$^{\$}$		
59	NCTC 13438	*K. pneumoniae*	$bla_{KPC\text{-}3}$<br>$bla_{OXA\text{-}9}^{\$}$<br>*aph(3')-Ia*$^{\$}$<br>*aac(6')-Ib* $^{\$}$<br>*aadA2b* $^{\$}$<br>*aadA2* $^{\$}$<br>*fosA* $^{\$}$<br>*mph(A)*$^{\$}$<br>*OqxA* $^{\$}$<br>*OqxB* $^{\$}$<br>*aac(6')-Ib-cr* $^{\$}$<br>*sul 1*$^{\$}$<br>*dfrA12* $^{\$}$<br>$bla_{SHV\text{-}182}^{\$}$<br>$bla_{\text{TEM-1A}}^{\$}$<br>*qacE* $^{\$}$<br>*catA1* $^{\$}$	bla_{KPC}: 19/19/16	⊚
60	NCTC 13476	*E. coli*	bla_{IMP}<br>*aac(3)-IId* $^{\$}$	bla_{IMP}: 21/21<br>$bla_{\text{IMP-1}}$: 20	⊚

⊚ = correlation; ? = unknown correlation; $ = genes not present on AMR-TAC; MRSA: methicillin resistant *Staphylococcus aureus; MSSA: methicillin susceptible Staphylococcus aureus;* VRE: vancomycin resistant *Enterococcus*⊚

Table S5: Concordance of antimicrobial resistance gene TaqMan array card results in faecal and respiratory samples obtained from children with suspected lower respiratory tract infection.

$bla_{CTX-M-1}$		Respiratory +	Respiratory -	
Faecal	+	0	1	1
Faecal	-	0	66	66
		0	67	

$bla_{CTX-M-9}$		Respiratory +	Respiratory -	
Faecal	+	0	1	1
Faecal	-	0	66	66
		0	67	

ermB		Respiratory +	Respiratory -	
Faecal	+	5	30	35
Faecal	-	5	27	32
		10	57	

mecA		Respiratory +	Respiratory -	
Faecal	+	3	6	9
Faecal	-	5	53	58
		8	59	

mecC		Respiratory +	Respiratory -	
Faecal	+	0	2	2
Faecal	-	0	65	65
		0	67	

vanB		Respiratory +	Respiratory -	
Faecal	+	0	1	1
Faecal	-	0	66	66
		0	67	

TOTAL		Respiratory +	Respiratory -	
Faecal	+	8	41	49
Faecal	-	10	353	363
		18	394	
